# Establishing a Cutoff Serum Thyroglobulin Value for the Diagnosis and Management of Well-Differentiated Thyroid Cancer

**DOI:** 10.1055/s-0043-1771286

**Published:** 2023-09-06

**Authors:** Jiwan Paudel

**Affiliations:** 1 Department of Nuclear Medicine, Chitwan Medical College, Bharatpur, Nepal

**Keywords:** thyroglobulin, radioiodine whole-body scintigraphy, differentiated thyroid cancer, cutoff

## Abstract

**Objective** The aim of this study was to define a cutoff serum thyroglobulin (Tg) level associated with either residual or metastasis that may help decide postoperative radioactive iodine (RAI) scan and treatment in differentiated thyroid cancer (DTC) patients residing in low-income countries like Nepal.

**Methods** We prospectively studied a total of 81 patients (female-to-male ratio of 3.0:1; mean age: 37.3 ± 14.0 years, within age range of 14–88 years) who underwent total thyroidectomy with/without neck dissection and were referred for RAI whole-body scan (WBS) ± RAI ablation or adjuvant treatment in the department of Nuclear Medicine, Chitwan Medical College. We calculated the cutoff value of Tg using receiver operating characteristic (ROC) curve analysis.

**Results** Forty-six of 81 patients (56.7%) had remnants in the thyroid bed, 26/81 (32.1%) had regional lymph node metastasis, 9/81 (11.1%) had distant lymph node metastasis, 3/81 (3.7%) had lung metastases, and only 1/81 (1.2%) had bone metastases. RAI WBS was positive in 61/81 (75.3%) patients and negative in 20/81 (24.7%) patients. Seventeen of 81 (20.9%) patients had negative RAI scans with low serum Tg levels; only 3/81 (3.7%) patients had Tg elevated negative RAI scan (TENIS). Although scan was positive in 61/81 (75.3%) patients, 64/81 (79.0%) patients received treatment with RAI, of which 3/81 (3.7%) patients were TENIS patients. There was a significant difference in serum Tg levels between patients who received or did not receive RAI ablation or treatment ( *p* < 0.05). On ROC curve analysis, the cutoff value of Tg levels between patients who received and did not receive treatment was 2.9 ng/mL (sensitivity: 85.9%; specificity: 94.1%; positive predictive value [PPV], 98.2%; negative predictive value [NPV]: 64.0%; AUC: 0.938).

**Conclusion** We identified a cutoff value of 2.9 ng/mL between patients who required or did not require treatment with high sensitivity, specificity, and PPVs.

## Introduction

Thyroglobulin (Tg) is a 660-kDa molecular weight glycoprotein synthesized by thyrocytes and released into the thyroid follicular lumen. 1 Tg production is stimulated by intrathyroidal iodine deficiency or excess, thyroid-stimulating hormone (TSH), and the presence of thyroid-stimulating immunoglobulins. 2 Several studies have investigated the role of preoperative serum Tg values as a predictor of malignancy in thyroid nodules. The American Thyroid Association (ATA), however, does not recommend routine preoperative measurement of serum Tg and Tg antibodies because there is no definite evidence that this has changed in patient management or outcome. The half-life of serum Tg is 1 to 3 days. Postoperative nadir is reached approximately 3 to 4 weeks after operation. 3 4 After radioactive iodine (I-131) therapy, it takes several months for Tg to disappear from the circulation. 5 The postoperative serum Tg value is an important prognostic factor that can guide patient management, especially in decision-making process for radioiodine ablation treatment and also predict successful ablation of the thyroid remnant. Postoperative serum Tg values greater than 10 ng/mL indicate the probability of persistent or recurrent disease, presence of distant metastases, failing I-131 ablation, and mortality, thus prompting additional evaluation and treatment. However, Tg level itself is not the only criterion for successful ablation, and patient risk group should also be considered.

Although postoperative stimulated serum Tg level above 10 to 30 ng/dL indicates worse prognosis, the value of Tg that is associated with either residual or metastasis is not yet defined. No optimal postoperative serum Tg cutoff level has been established to help determine the indication for radioactive iodine (RAI) ablation in the ATA guidelines. 6 However, some authors advocate that radioiodine scan and ablation can be completely omitted in patients with low postoperative stimulated Tg (≤ 1 ng/mL) and nonstimulated Tg levels (0.2–0.3 ng/mL) with negative antithyroglobulin antibodies (anti-TgAb). 7 8 9 Thus, these patients need no cutoff of Tg level. However, physicians and surgeons in Nepal are specially concerned if a patient shows a higher Tg level. They have to decide whether they recommend RAI scan or direct RAI treatment based on Tg level because medical equipment such as ultrasonography or computed tomography are not available in the majority of hospitals in remote areas in Nepal. In these cases, the cutoff level of Tg is considerable specially in the context of Nepal with lack of medical equipment in the majority of hospitals and poor socioeconomic status of patients. Patients have to travel several days for nuclear medicine facility, creating a huge economic impact in low-income countries like Nepal.

Mourão et al concluded that papillary thyroid carcinoma patients with low nonstimulated Tg levels (Tg < 0.3 ng/mL) and negative nodal status in the neck after thyroid surgery did not require postoperative I-131 treatment. 10 These patients need no cutoff of Tg level. Thus, our study aimed to define a cutoff-stimulated Tg level in patients with elevated Tg level that may help guide for RAI scan and treatment.

## Material and Methods

A total of 81 patients comprising 20 males and 61 females (female-to-male ratio of 3.0:1 and mean age of 37.3 ± 14.0 years, within the age range of 14–88 years) who underwent total thyroidectomy with/without neck dissection at various hospitals from all over Nepal and were referred for whole-body scan (WBS) ± RAI ablation in the Department of Nuclear Medicine, Chitwan Medical College, during the period from December 2020 to December 2021 were selected prospectively for the study. Serum Tg and TgAbs were measured in all patients. Although there are various techniques for measuring Tg levels, the electrochemiluminescence immunoassay (ECLIA) intended for use on **cobas e** immunoassay analyzers for the in vitro quantitative determination of Tg in human serum and plasma was used in this study. Similarly, TgAb determination was also done with ECLIA intended for use on **cobas e** immunoassay analyzers for the in vitro quantitative determination of antibodies to Tg in human serum and plasma

All patients underwent WBS and RAI ablation or adjuvant treatment was performed in patients with either residual or metastasis. A diagnostic WBS was conducted using a dual-head gamma camera (Siemens Intevo Bold single photon emission tomography/computed tomography [SPECT/CT]). Prior to the scan, patients were instructed to stop taking thyroxine for a period of 3 to 6 weeks. None of the patients were administered Thyrogen since the study was conducted in a low-income country. Target TSH for diagnostic scan and/or ablation was greater than 30 ng/mL. Patients were kept at a low-iodine diet for a period of approximately 10 days before imaging. Low oral diagnostic dose in the range of 1.5 to 2.5 mCi was used for imaging to avoid the stunning effect of the high administered dose. Imaging was performed approximately 48 hours after the administered dose. Wide field of view gamma camera with computer acquisition along with high-energy parallel hole collimator with 20% window centered at 364 keV was used for acquisition.

The table speed was set at 8 cm/min and a matrix size of 256 × 1,024 was used. All patients underwent planar WBS imaging protocol, with anterior and posterior views. Additional anterior and posterior views of other anatomical body parts were obtained based on the findings of the WBS planar image results. The SPECT/CT imaging was performed immediately post-WBS. The SPECT/CT of the neck and chest regions was routinely acquired for all patients after WBS planar imaging. Based on WBS planar findings, extra SPECT/CT views were performed for other anatomical parts of the body, such as the skull, abdomen/pelvis, or extremities. SPECT views were obtained in a step-and-shoot sequence (25 s/stop), with 64 frames/head obtained using a noncircular orbit, over 360 degrees (180 degrees per head); tomographic views were reconstructed into a 128 × 128 matrix using iterative reconstruction (IR). CT was used for attenuation correction, and anatomical localization was obtained with a tube voltage of 130 kV; the tube current was 70 mAs. The reconstructed slice width was 2 mm.

Reading of the RAI scan was performed by an expert, senior Nuclear Medicine Physician, and decision for treatment was made after discussion with the referring surgeon. The results of planar WBS images were considered positive when single or multiple areas of uptake inconsistent with physiologic activity were recognized. Diffuse uptake was considered normal physiological uptake in the liver, gastrointestinal tract, and urinary bladder. The result of SPECT/CT image was considered positive when single or multiple areas of uptake with or without CT correlate were recognized after exclusion of physiologic uptake. The findings of both planar WBS and SPECT/CT scans were confirmed by clinical assessments, the primary routine investigation by neck ultrasound (US) scan, and Tg and TgABs.

RAI remnant ablation or adjuvant treatment was performed in patients with either residual or metastasis. A minimum of 2 months' time interval was maintained between iodinated contrast CT scan performed preoperatively and WBS or treatment. All patients with contraindications to the use of RAI like pregnant and lactating women were denied RAI scan and thus were not enrolled in the study. Patients with negative RAI scans but with elevated serum Tg levels also received empirical RAI treatment in the range of 100 mCi and were included in the study. RAI ablation or adjuvant treatment dose was in the range of 50 to 200 mCi at a single setting. In remnants, 50 mCi was used, 100 mCi was used when lymph nodes (LNs) were involved, 150 mCi was used if the lung was involved, and 200 mCi was used if bones were involved. A maximum total cumulative dose of 800 mCi was used for bone metastasis. All ablations or adjuvant treatments were performed on inpatient basis admitting in isolated therapy wards for 1 to 4 days until the radiation level was set at discharge limit. Strict radiation safety precautions were undertaken during therapy and patients were instructed both in verbal and written form for radiation safety precautions for a period of at least a week following RAI therapy.

## ROC Curve and Statistical Analysis

An optimal cutoff value of Tg indicating whether RAI ablation or adjuvant treatment was needed or not needed was calculated using the receiver operating characteristic (ROC) curve analysis. The optimal cutoff of Tg level was also calculated for the scan being positive or negative, for residual only, for LN metastasis, and for lung and bone metastases. Thus, overall diagnostic performance of Tg level was evaluated using an ROC curve analysis.

## Results

**Fig. 1:**
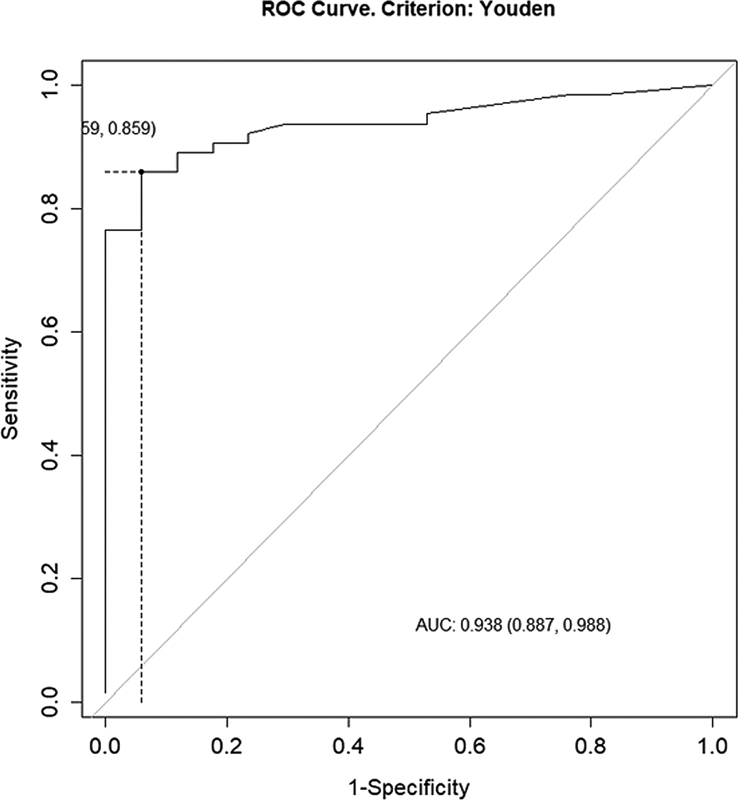
Receiver operating characteristic (ROC) curve between patients who received and did not receive treatment. AUC, area under curve.

**Fig. 2:**
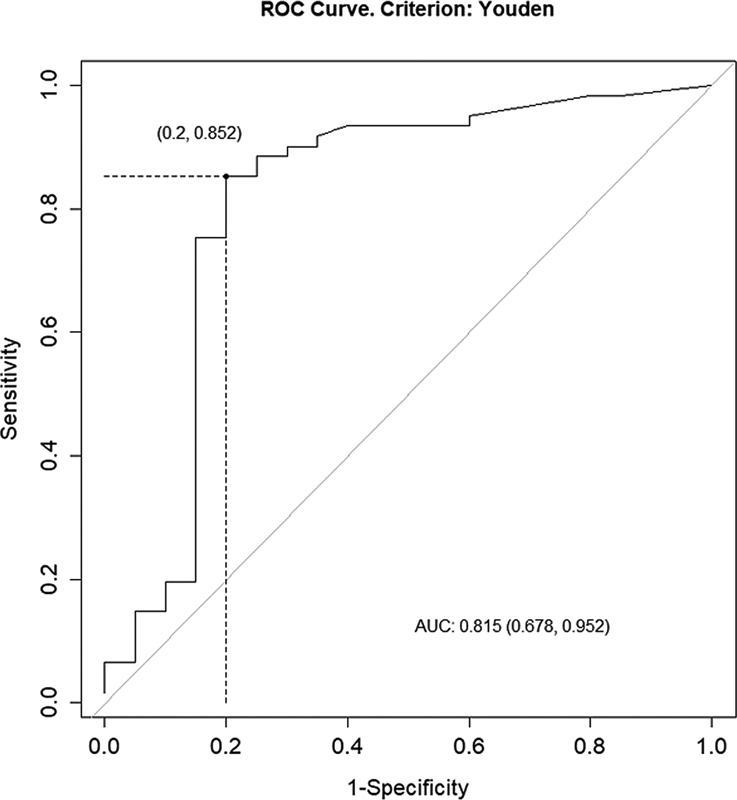
ROC curve for the scan being positive or negative. AUC, area under curve.

We chose 81 patients who had undergone total thyroidectomy with or without neck dissection and had been referred for a WBS RAI, with or without RAI ablation or adjuvant treatment, from multiple hospitals throughout Nepal during the study period. In all, 46/81 patients (56.7%) had remnants in the thyroid bed, 26/81 (32.1%) had regional LN metastasis, 9/81 (11.1%) had distant LN metastasis, 3/81 (3.7%) had lung metastases, and only 1/81 (1.2%) had bone metastases. RAI WBS was positive in 61/81 (75.3%) patients and negative in 20/81 (24.7%) patients. In 17/81 (20.9%) patients RAI scans were negative with low serum Tg levels and thus were followed up with thyroid hormone replacement therapy. Only 3/81 (3.7%) patients had a negative RAI scan with elevated Tg levels. These patients were labeled as Tg elevated negative iodine scan (TENIS) and thus were also treated with empirical RAI therapy ( Fig. 1 ). Although scan was positive in 61/81 (75.3%) patients, 64/81 (79.0%) patients received treatment with RAI, out of which 3/81 (3.7%) patients were TENIS patients. There was a significant difference in serum Tg levels between patients who received and did not receive RAI treatment ( *p* < 0.05). On ROC curve analysis, the cutoff value of Tg levels between patients who received and did not receive treatment was 2.9 ng/mL (sensitivity: 85.9%; specificity, 94.1%; positive predictive value [PPV]: 98.2%; negative predictive value [NPV]: 64.0%; AUC: 0.938; Fig. 2 ).

**Fig. 3:**
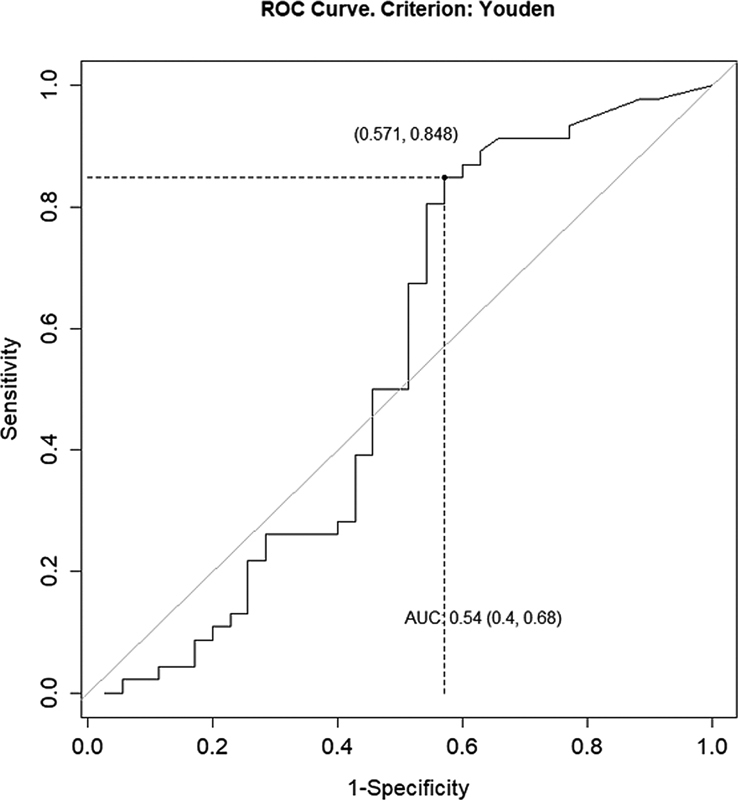
ROC curve for residuals only. AUC, area under curve.

**Fig. 4:**
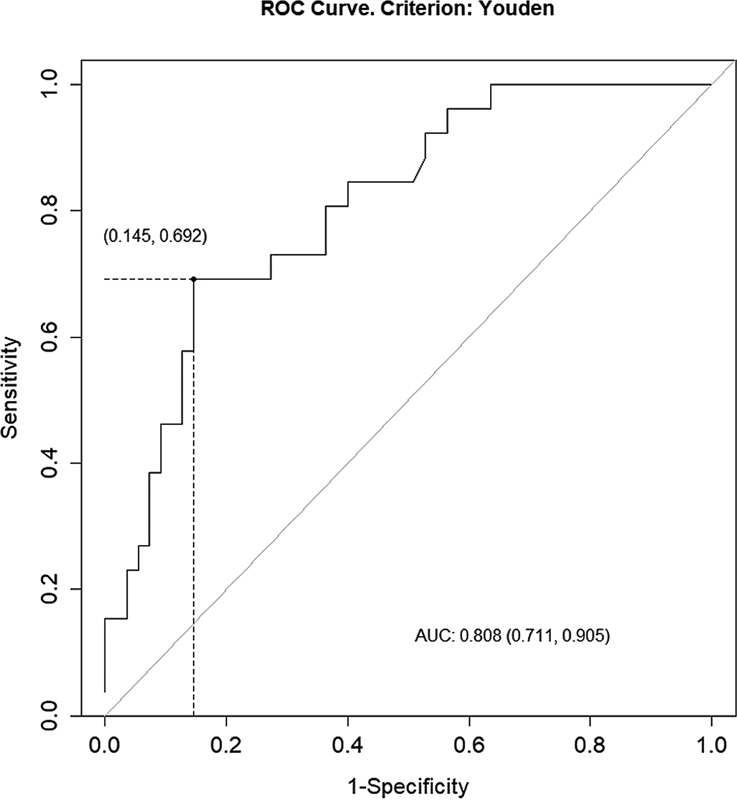
ROC curve for lymph node metastasis. AUC, area under curve.

**Fig. 5:**
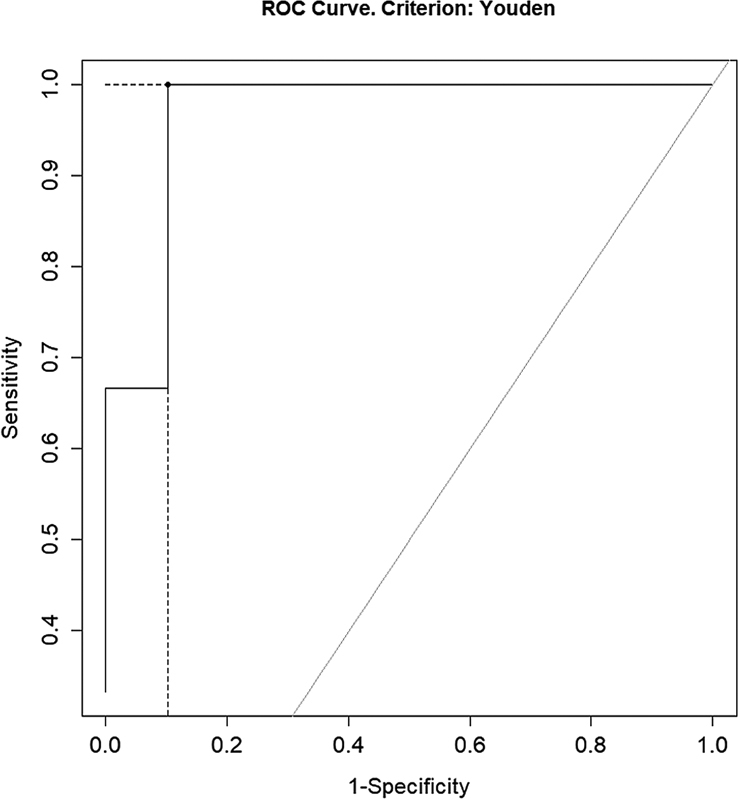
ROC curve for lung and bone metastases.

The higher level of Tg more accurately predicted a high risk of remnant or metastasis ( Figs. 3 and 4 ). The cutoff value was also 2.9 ng/mL for the scan being positive or negative (sensitivity: 85.2%; specificity: 80.0%; PPV: 92.8%; NPV: 64.0%; AUC: 0.815; Fig. 5 ).

**Fig. 6:**
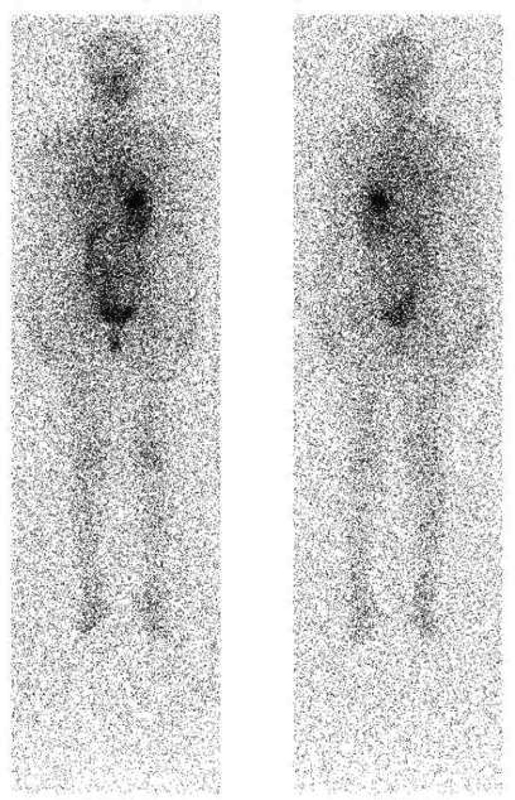
TENIS: Scan negative; Tg: 73.3 ng/ml.

**Fig. 7:**
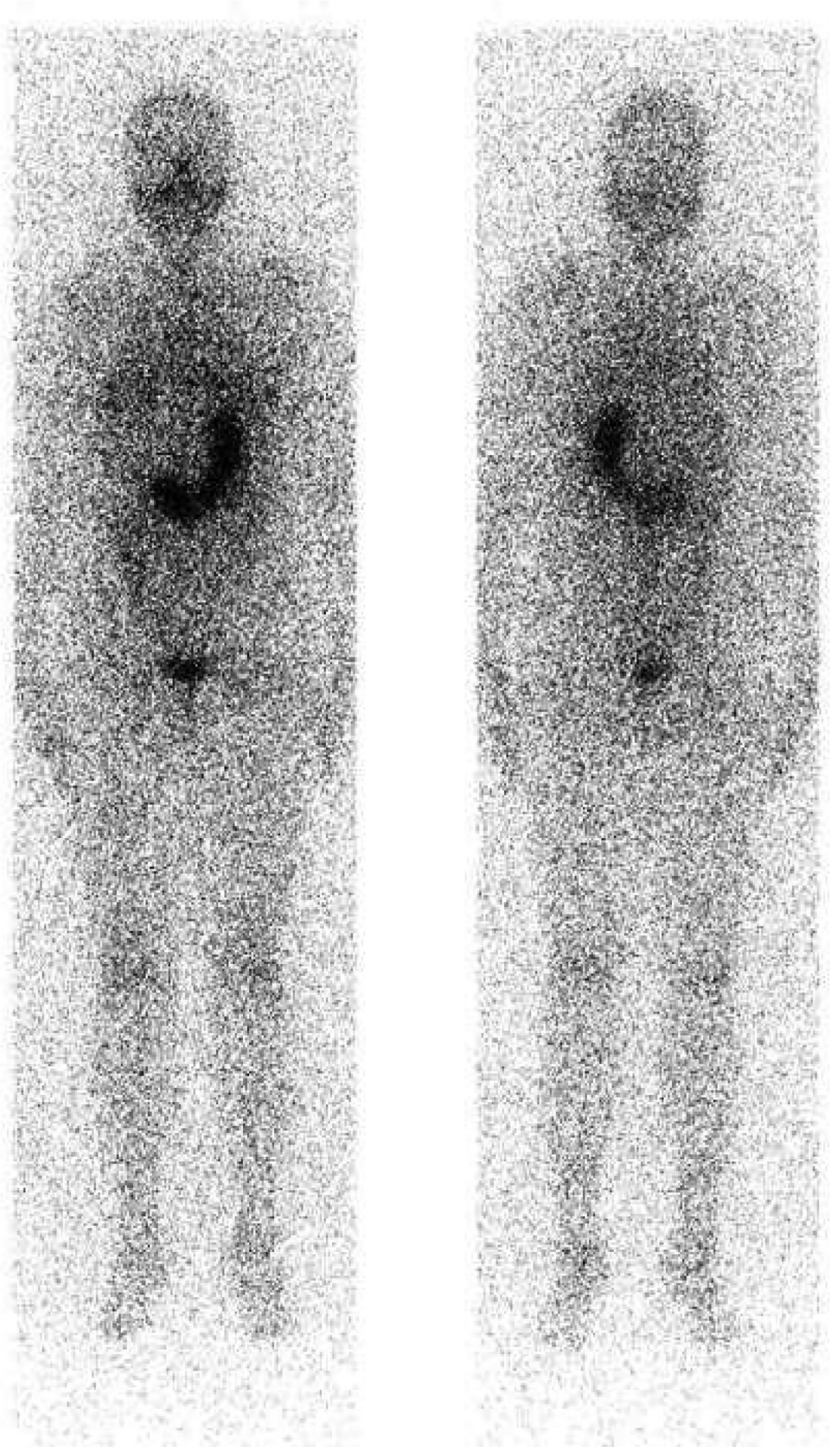
Negative scan; Tg: 0.01 ng/ml.

**Fig. 8:**
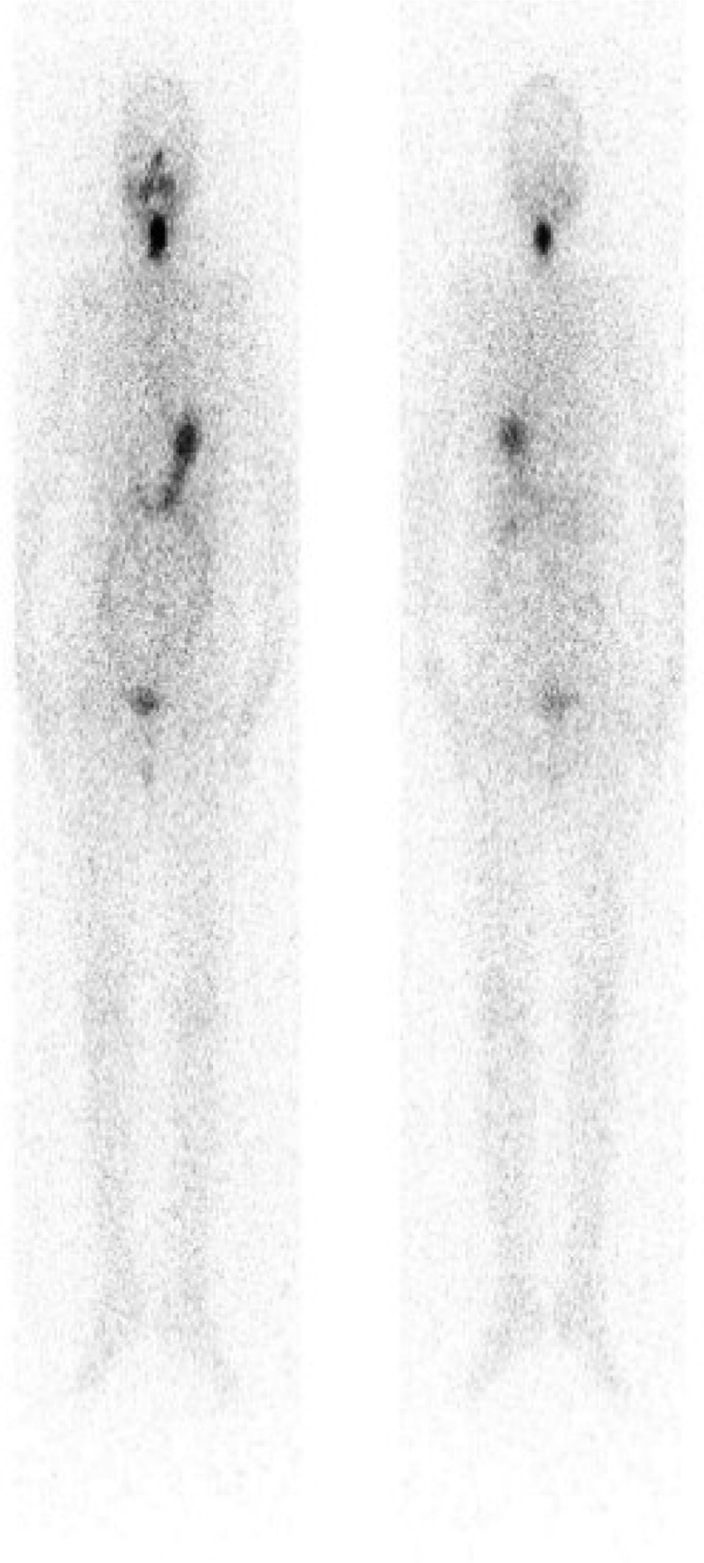
Residual; Tg: 16.9 ng/ml.

We identified a cutoff value of 1.8 ng/mL for remnant only (sensitivity: 84.7%; specificity: 42.8%; PPV: 66.1%; NPV: 68.1%; AUC: 0.54; Figs. 6 and 4 ), a cutoff of 37.0 ng/mL for LN metastasis (sensitivity: 69.2%; specificity: 85.4%; PPV: 69.2%; NPV: 85.4%; AUC: 0.808; Fig. 7 ), and a cutoff of 85.6 ng/mL for lung and bone metastases (sensitivity: 100%; specificity: 89.7%; PPV: 27%; NPV: 100%: AUC: 0.966; Fig. 8 ).

## Discussion

Thyroid cancer accounts for approximately 1% of all cancers. However, it is the most common type of endocrine malignancy. 11 12 Differentiated thyroid cancers (DTCs) account for 90% of all thyroid cancers. They are associated with low malignant potential and a very good prognosis. 13 The majority of patients with DTC are treated initially with total thyroidectomy with or without neck dissection. However, lobectomy may be performed in patients with microcarcinoma without nodal metastases on imaging. 14 15 Postoperative I-131 ablation or adjuvant treatment is indicated in all high-risk patients; however, there is no overall agreement regarding its indication in low- and intermediate-risk patients. 16 17 18 Our study aimed to determine a threshold serum Tg level that is linked to either remaining cancerous cells or metastasis. This could assist in deciding whether to conduct a postoperative RAI scan and treatment for DTC, particularly for patients with limited financial means living in low-income countries such as Nepal, where traveling for several days to undergo RAI scan and therapy is necessary.

RAI whole-body scintigraphy and treatment is routinely performed in most of the developed countries following total thyroidectomy because it is widely available and may not have significant economic burden to patients because in the majority of countries it is under insurance coverage. However, in low-income countries like Nepal, where RAI whole-body scintigraphy is available in only two centers as of 2022, and where patients have to travel several days to reach the facility, low-income patients may not be in a position to bear all economic burden. Serum Tg levels are important in patients from low- and intermediate-risk groups, where the selection of patients for adjuvant therapy can be made based on Tg values. Interestingly, some studies have shown the correlation of postoperative serum Tg levels with success of remnant ablation demonstrating the higher rates of radioiodine ablation failure with Tg values greater than 5 to 6 ng/mL regardless of RAI dose used for treatment. 19 20

Since Tg is secreted by the follicular cells of the thyroid, several studies have demonstrated correlation of high postoperative serum Tg values with the increased risk of recurrence or persistent disease during follow-up after total thyroidectomy and RAI ablation. 21 22 23 24

We selected 81 patients from different hospitals across Nepal who had undergone total thyroidectomy, with or without neck dissection, and were referred for a whole-body RAI scan with or without RAI ablation or adjuvant therapy during the study period. Some studies have demonstrated the predictive value of postoperative serum Tg levels for detecting distant metastasis, but these studies have typically excluded patients with only cervical lymph nodal metastases. 25 26 27 28 Our study aimed to establish a cutoff value for serum Tg levels in patients residing in low-income countries, so that if the level falls below this cutoff value, iodine whole-body scintigraphy could be avoided, thus reducing the unnecessary economic burden and travel for patients residing in low-income countries like Nepal.

In this prospective study, we selected a total of 81 patients (20 males and 61 females; female-to-male ratio of 3.0:1) with a mean age 37.3 ± 14.0 years (range: 14–88 years). All patients underwent total thyroidectomy with/without neck dissection and were referred from various hospitals all over Nepal for whole-body RAI scan ± RAI ablation or adjuvant treatment in the Department of Nuclear Medicine, Chitwan Medical College, during the period from December 2020 to December 2021.

In total, 46/81 patients (56.7%) had remnants in the thyroid bed, 26/81 (32.1%) had regional LN metastasis, 9/81 (11.1%) had distant LN metastasis, 3/81 (3.7%) had lung metastases, and only 1/81 (1.2%) had bone metastases. Only 3/81 (3.7%) patients had negative RAI scan with elevated Tg levels (TENIS). The antithyroglobulin levels in these patients were within normal limits. Ultrasonography performed in all of these patients showed few subcentimetric cervical nodes, globular in outline with loss of fatty hilum, and not amenable for resection. Thus, we confirmed that Tg levels were not falsely elevated and labeled them as TENIS and treated accordingly with available resources. A study by Jiwang et al showed the frequency of central lymph nodal and lateral compartmental lymph nodal metastases in DTC to be 22.4 and 10.0%, respectively, which closely matches our study findings of regional lymph nodal metastasis of 32.1% because our study included both central lateral compartments in regional lymph nodal metastases. 29 A study by Iñiguez-Ariza et al showed incidence of osseous metastasis in thyroid cancer to be 4%, while our study showed only 1%. 30 This difference is attributed to quite small sample size of the study and the fact that osseous metastasis in thyroid cancer is a poorly studied entity. Another study by Kalender et al showed the incidence of lung metastases to be 3.1%, which is in close resemblance to our study. 31 However, we suggest further studies with a larger sample size to verify our results. We did not, however, find any study that directly shows the incidence of remnant after surgery in DTC. These data, however, are likely to differ in different institutions based on the expertise of the treating surgeon.

We found a significant difference in serum Tg levels between patients who received or did not receive RAI treatment ( *p* < 0.05). On ROC curve analysis, the cutoff value of Tg levels between patients who received and did not receive treatment was 2.9 ng/mL (sensitivity: 85.9%; specificity: 94.1%; PPV: 98.2%; NPV: 64.0%; AUC: 0.938; Fig. 2 ). The cutoff value was also 2.9 ng/mL for the scan being positive or negative (sensitivity: 85.2%; specificity: 80.0%; PPV: 92.8%; NPV: 64.0%; AUC: 0.815; Fig. 5 ). The higher level of Tg more accurately predicted a high risk of remnant or metastasis. However, we did not find any studies in the literature that identified a cutoff value suggestive of remnant or a metastasis requiring treatment or a scan being positive or negative.

**Fig. 9:**
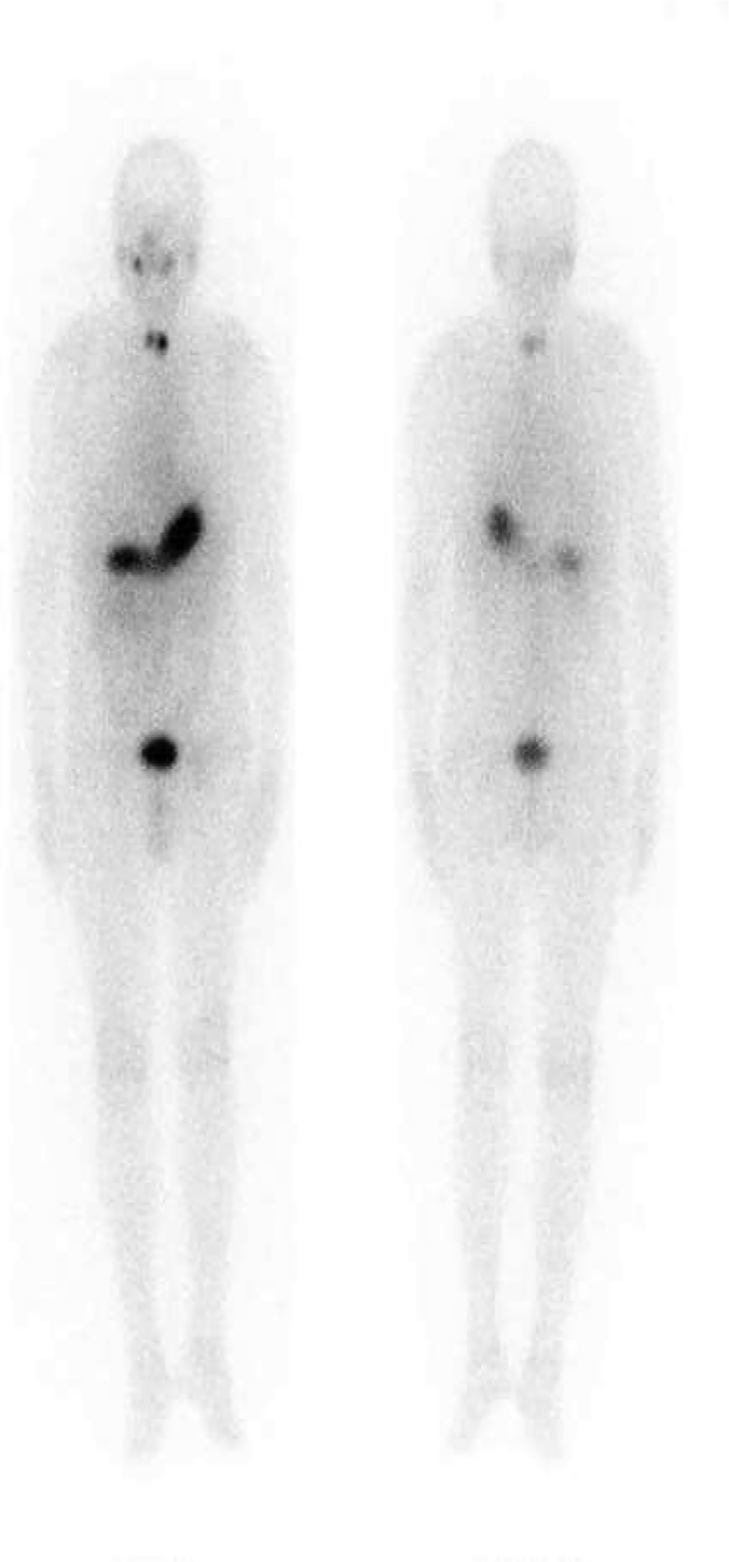
Lymph node metastases; Tg: 84.6 ng/ml.

**Fig. 10:**
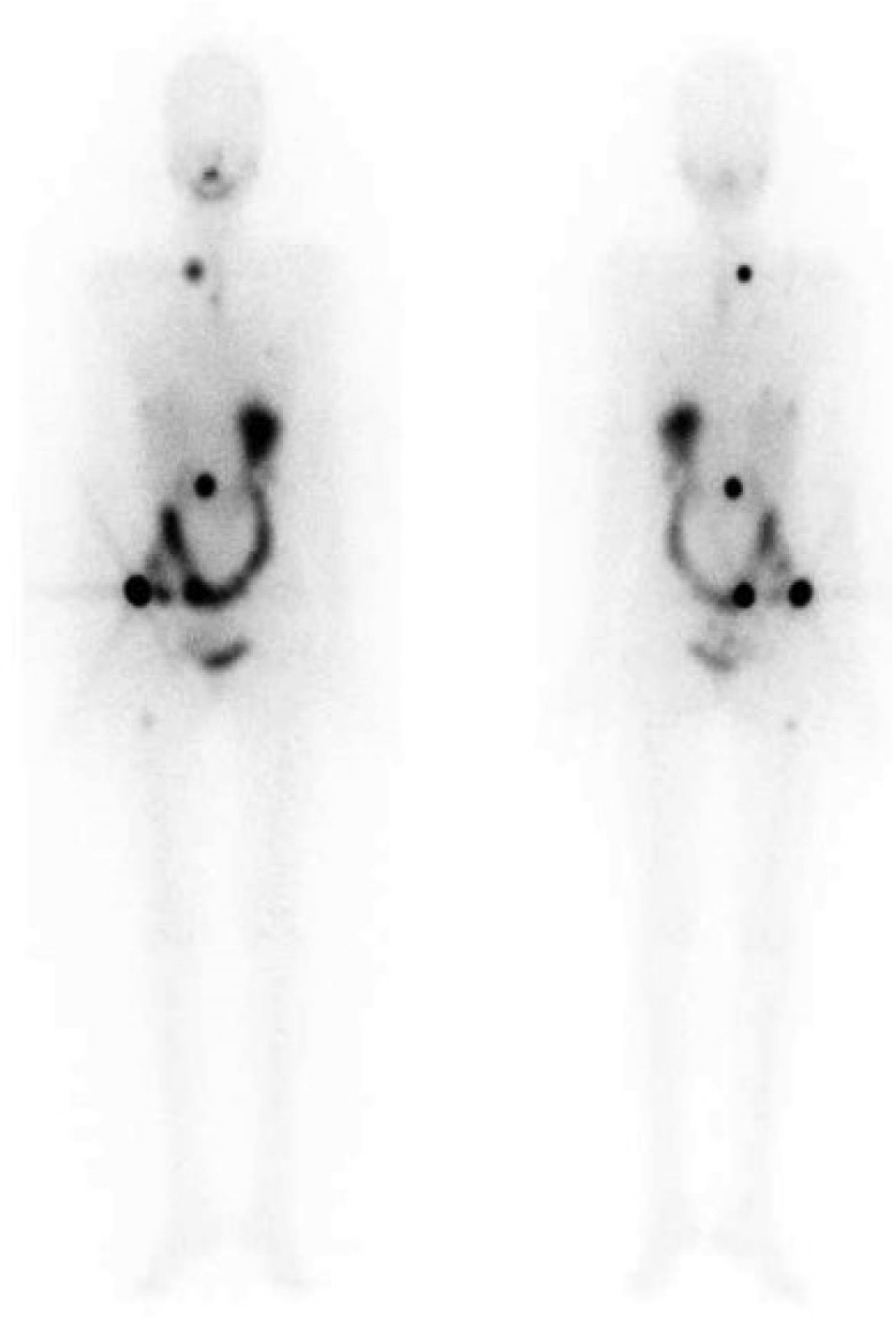
Lung and bone metastases; Tg: 1,980 ng/ml.

We identified a cutoff value of 1.8 ng/mL for remnant only (sensitivity: 84.7%; specificity: 42.8%; PPV: 66.1%; NPV: 68.1%; AUC: 0.54; Fig. 6 ), a cutoff of 37.0 ng/mL for LN metastasis (sensitivity: 69.2%; specificity: 85.4%; PPV: 69.2%; NPV: 85.4%; AUC: 0.808; Fig. 9 ), and a cutoff of 85.6 ng/mL for lung and bone metastases (sensitivity: 100%; specificity: 89.7%; PPV: 27%; NPV: 100%; AUC: 0.966; Fig. 10 ).

Although we did not find studies identifying cutoff values of serum Tg postoperatively, Zhai et al identified the optimal cutoff value of Tg in fine-needle aspiration (FNA-Tg) in distinguishing benign LNs from malignant LNs as 19.4 ng/mL (sensitivity: 85.3%; specificity: 93.8%; AUC: 0.94 [95% confidence interval [CI]: 0.91–0.96]) in all cases. 32 The optimal cutoff value was higher in the group with thyroid tissue than those without thyroid tissue. In the group with thyroid tissue, the optimal cutoff value was still 19.4 ng/mL (sensitivity: 93.4%; specificity: 92.9%; AUC = 0.95; 95% CI: 0.92–0.97). In the group after total thyroidectomy, the optimal diagnostic threshold was 1.2 ng/mL (sensitivity: 96.1%; specificity: 87.0%; AUC = 0.93; 95% CI: 0.85–0.98). The difference in values of Tg is related to the fact that we studied serum Tg levels, but other studies studied FNA-Tg levels.

Couto et al identified the cutoff serum Tg level of 117.5 ng/mL, suggestive of distant metastasis with a high NPV of 93.7%. 11 This cutoff value was for lung and bone metastases. Our study showed a cutoff value of 85.6 ng/mL for lung and bone metastases. Thus, there is a significantly high level of Tg for bone and lung metastases as observed in both these studies. The difference in Tg level in between these studies is related to the fact that there were very few cases with lung and bone metastases in our study. Thus, further studies with a larger number of bone and lung metastases are needed to verify these findings.

## Conclusion

Our findings demonstrated that serum Tg could predict either remnant or metastases requiring treatment with RAI in DTC patients. We identified a cutoff value of 2.9 ng/mL between patients who required or did not require treatment (sensitivity: 85.9%; specificity: 94.1%, with a high PPV of 98.2%) with RAI. Thus, in patients residing in low-income countries like Nepal, postoperative RAI scan and treatment should be performed in patients with Tg ranges higher than 2.9 ng/mL and may be avoided in Tg ranges lower than the cutoff level in selected patients.
